# Spatial analysis between particulate matter and emergency room visits for conjunctivitis and keratitis

**DOI:** 10.1186/s40557-018-0252-x

**Published:** 2018-06-11

**Authors:** Jung-Youb Lee, Jung-Won Kim, Eun-Jung Kim, Mi-Young Lee, Chang-Wook Nam, In-Sung Chung

**Affiliations:** 1 Division of Occupational and Environmental Medicine, Keimyung University Dongsan Medical Center, Daegu, South Korea; 2 Department of Urban Planning, Keimyung University School of Engineering, Daegu, South Korea; 3 Department of Preventive Medicine, Keimyung University School of Medicine, Daegu, South Korea; 4 Division of Cardiology, Keimyung University School of Medicine, Daegu, South Korea

**Keywords:** Particulate matter, PM_10_, Conjunctivitis, Keratitis, Spatial analysis

## Abstract

**Background:**

The concentration of particulate matter in the air varies depending on the region because it is lightweight and generated from a variety of sources. To assess the relationship between particulate matter and eye disease, this study analyzes the concentration data obtained from spatial analysis of particulate matter and emergency visit data.

**Methods:**

The study included 769 residents of Daegu, Korea who had visited an emergency room for the problem of conjunctivitis or keratitis. Concentrations of PM_10_ and other air pollutants were obtained from the Korean Ministry of the Environment. PM_10_ concentrations and the number of patients from each of 143 administrative dongs (sub-municipal level administrative units) of the city of Daegu were obtained using spatial analysis. The patient distribution and PM_10_ concentration were mapped for comparison, and their relationship was examined using scatter plot, regression analysis, and the independent sample t-test.

**Results:**

The number of patients with conjunctivitis and keratitis was significantly higher in the regions of the top 20% areas than the bottom 20% areas in terms of PM_10_ concentration. The distribution of PM_10_ concentration and number of patients was visually similar on the map. The concentration of PM_10_ and the number of patients showed a dose–response relationship. When the concentrations of other air polluta9nts were controlled for, the numbers of conjunctivitis and keratitis patients were 0.04 per 1000 ER patients and 0.10 per 1000 ER patients, respectively.

**Conclusion:**

As PM_10_ is associated with the prevalence of conjunctivitis and keratitis, measures to reduce particulate matter through environmental methods are needed.

**Electronic supplementary material:**

The online version of this article (10.1186/s40557-018-0252-x) contains supplementary material, which is available to authorized users.

## Background

Exposure to particulate matter has been reported to contribute to various illnesses, such as pulmonary disease, cardiovascular disease, cerebrovascular disease, and eye disease [1–9]. Sources of particulate matter vary widely, including road dust, resuspended sediment, fossil fuel burning, sea salt, automobile exhaust gas, wood burning, ships, railroad tracks, biomass burning, industrial processes, and cooking [10]. Consequently, the amount of particulate matter generated varies across regions, and because it moves constantly owing to its lightweight, its concentration shows spatial variation depending on the geographic location, such as urban and rural areas, seashores, inland areas, near highways, communities, and areas with ascending and descending air flow [11]. Thus, research has recently been conducted on the effects of particulate matter on health, considering the spatial variation across different regions rather than studying a single area. A U.S. study investigated the relationship between the amount of PM_2.5_ exposure calculated based on residence address and morality [12]; a German study examined the association between the amount of PM_2.5_ exposure, which was calculated based on the distance between the residence and the road, and cognitive disorder status [13]; a study conducted in Los Angeles showed the relationship between PM_2.5_ concentration and mortality using spatial analysis [14].

Other studies have investigated the relationship between particulate matter and eye disease. It has been reported that high levels of PM_10_ may increase the number of outpatients with conjunctivitis [15], and that high levels of PM_2.5_ may cause keratitis [16]. However, little research has been conducted on the relationship between particulate matter and eye disease considering the spatial variation.

Regarding the study area in the present work, the city of Daegu was selected as it has a large geographical range, sufficient amount of exposure to air pollution, and objective air pollution and patient data are available. Daegu is the fourth most populous city of Korea and has the third highest number of vehicles of cities in Korea [17]. Daegu has 13 air quality monitoring stations [18], and its monthly average PM_10_ concentration obtained based on 1996–2010 data was 62.2 ± 17.3 μg/m^3^, which was second highest next to Seoul [17]. Therefore, the present study investigates the relationship between the distribution of PM_10_ and the distribution of outpatients with conjunctivitis and keratitis using spatial analysis considering the geographical variation in Daegu.

## Methods

### Study population

The study was conducted with 769 Daegu residents who has visited the emergency department of Dongsan Medical Center for emergency treatment between June 1, 2006 and December 31, 2014, and were diagnosed with conjunctivitis or keratitis at the time of their visit. The patient diagnosis was based on patient disease information submitted to the National Emergency Medical Center, and conjunctivitis and keratitis were defined as H10.0–10.9 and H16.0–16.9, as specified in the Korean Standard Classification of Diseases (KCD) 7th revision. Patient residence was determined based on the addresses patients provided at the time of their visit to the emergency room, and all addresses were classified into one of 143 administrative dongs in Daegu as of 2005. This study was approved by the Institutional Review Board of the Dongsan Hospital of Keimyung University (IRB No. DSMC 2016–10-003).

### Exposure assessment

**Fig. 1:**
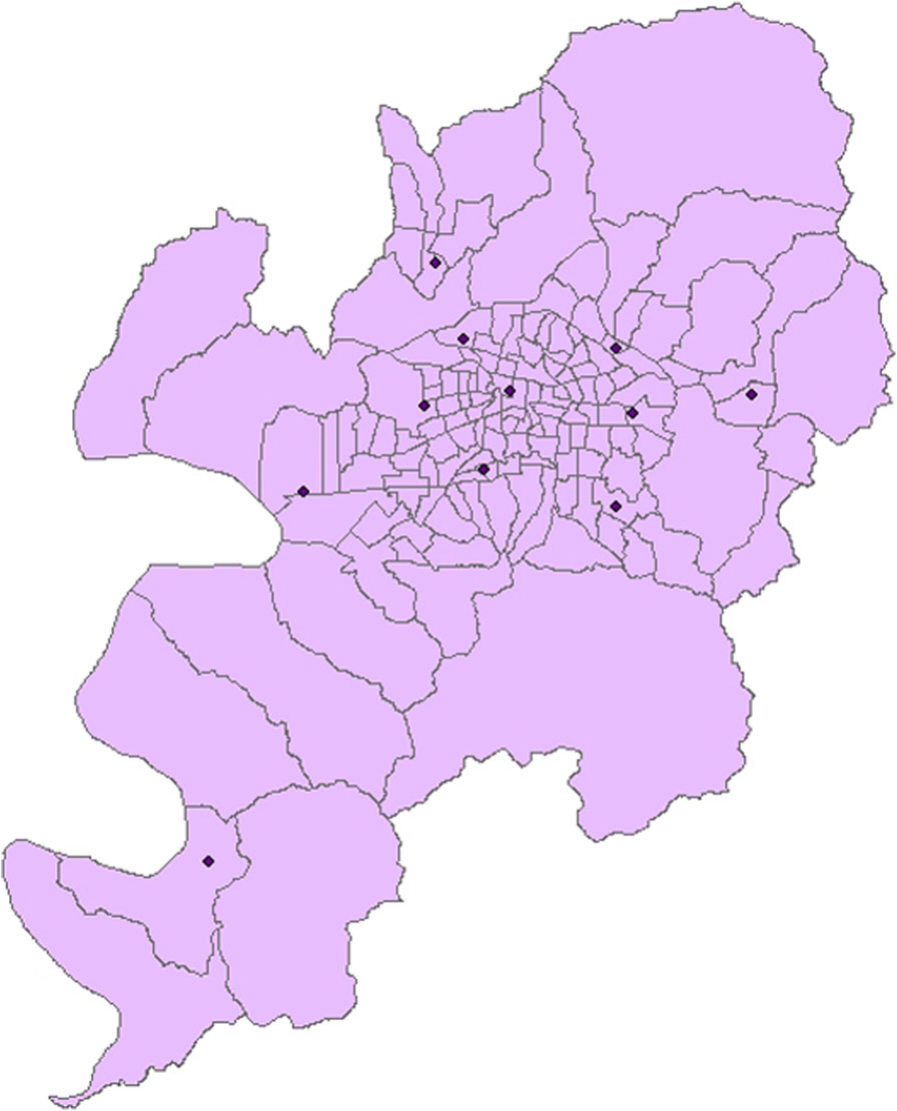
Location of air monitoring stations in each region

PM_10_ concentrations were calculated using the data from 11 air quality monitoring stations in Daegu, which were installed and operated by the Korean Ministry of the Environment and in which measurements are taken throughout the study period (Fig. 1). The concentration of PM_10_ was measured using the β-ray absorption method (BAM), an official method used in the air quality monitoring network [19]. The measurement unit was μg/m^3^ and the measurements were made at the integer level with no decimal points.

**Fig. 2:**
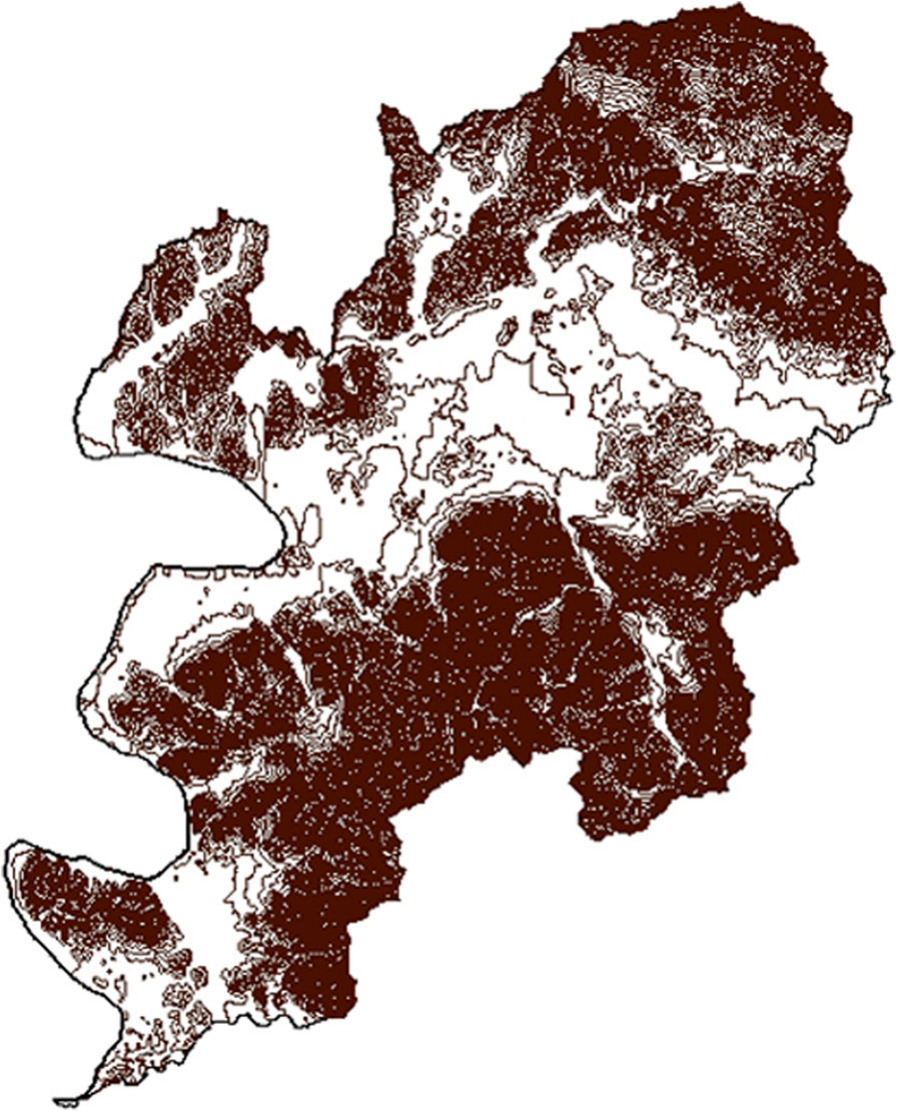
Polyline of Daegu’s contour line

In this study, spatial analysis requires the PM_10_ concentration for each administrative dong. However, there were 143 administrative dongs in Daegu in 2005, while there were only 11 monitoring stations in Daegu to use in the study. Moreover, the stations do not accurately measure the PM_10_ of the administrative dong in which they are located. Thus, the study used a geographic information system (GIS) to obtain more reliable PM_10_ concentrations for each administrative dong. First, PM_10_ concentrations for the areas without stations were calculated using inverse distance weighting (IDW) interpolation and the locations of monitoring stations in Daegu, which were obtained in the form of transverse Mercator coordinates from Daegu Health and Environmental Research Institute. Interpolation is a method used to estimate the values in the intervening space. IDW is known to be a flexible and popular method of interpolation [20]. The distribution of particulate matter is influenced by vertical variations in geography, such as elevation, which indicates how high the ground is above sea level, as well as horizontal variation in geography, such as administrative dongs [21]. Therefore, the present study incorporated the variation in elevation by co-kriging contour data for Daegu for additional interpolation when calculating concentrations of PM_10_ (Fig. 2). This suggests that, for the areas at horizontally the same distance from a specific station, the concentrations of PM_10_ are less likely to reflect the values measured at the station when there is more variation in elevation in the terrain between two sites because this increases the actual distance.

The PM_10_ measurements obtained using the process were rasterized, and the average concentration was obtained for each administrative dong. All spatial analyses were performed using ArcGIS 10.4.1 for desktop.

The data on the monthly average concentration of the four substances from June 2006 to December 2015 were obtained from the Ministry of the Environment. The air pollutant concentrations for each administrative dong to use in spatial analysis were calculated using GIS as in the calculation of PM_10_ concentrations. The measurement unit was ppm, and measurement was made to three decimal places for SO2, NO2, O3, and to one decimal place for CO.

### Statistical analysis

The analysis of the relationship between the average concentration of PM_10_ and the distribution of patients in the 143 administrative dongs in Daegu was conducted for each year. The Visit Index was used to determine the distribution of patients by administrative dong, and it was defined as the number of patients with conjunctivitis or keratitis per 1000 visitors to the emergency room of Dongsan Medical Center. An independent sample t-test was performed to determine whether the top 20% and bottom 20% areas in terms of PM_10_ concentrations significantly differed in prevalence of the diseases. Spatial analysis of mapping the distributions of PM_10_ and patients was performed using ArcGIS. Scatter plots were also used to determine whether PM_10_ concentration and the number of patients have a dose–response relationship. The association between PM_10_ and the number of patients was analyzed using simple linear regression analysis, which examined the effect of PM_10_ only, and multiple regression analysis, which examined both PM_10_ and other air pollutants. All statistical analyses were carried out at the 0.05 significance level using IBM SPSS Statistics 23.

## Results

The total number of study participants was 769, of whom 191 (24.8%) had conjunctivitis and 578 (75.2%) had keratitis. Regarding gender, 494 (64.2%) were male and 275 (35.8%) were female. Among the age groups divided into 20-year ranges, 268 (34.9%) were aged from 40 to less than 60, and 266 (34.6%) were aged from 20 to less than 40. Regarding the residence district, Seo-gu had most participant residents of 236 (30.7%) followed by Buk-gu with 205 (26.7%). The total number of visitors to the emergency room was 282,017. This number is equal to the sum of all the Visit Index denominators (Table 1).

**Table 1:** General characteristics of study subjects

Variables	Number (%)
Total	769 (100.0)
Conjunctivitis	191 (24.8)
Keratitis	578 (75.2)
Sex	
Male	494 (64.2)
Female	275 (35.8)
Age (years)	
< 20	157 (20.4)
20–40	266 (34.6)
40–60	268 (34.9)
60–80	75 (9.8)
≥ 80	3 (0.4)
Address (district)	
Nam-gu	26 (3.4)
Dalseo-gu	163 (21.2)
Dalsung-goon	31 (4.0)
Dong-gu	24 (3.1)
Book-gu	205 (26.7)
Seo-gu	236 (30.7)
Suseong-gu	44 (5.7)
Joong-gu	40 (5.2)
Total visitors (denominator)	282,017

Between June 2006 and December 2014, the average PM_10_ concentration was 48.15 μg/m^3^, the minimum was 27 μg/m^3^, and the maximum was 86 μg/m^3^. The mean concentrations of other air pollutants were 0.005 ppm for SO2, 0.024 ppm for NO2, 0.024 ppm for O3, and 0.5 ppm for CO (Table 2).

**Table 2:** Monthly average PM10, other air pollutant concentrations from June, 2006 to December, 2014

	Min	Max	Mean	SD
PM10 (μg/m^3^)	27	86	48	11.92
SO2 (ppm)	0.002	0.010	0.005	0.002
NO2 (ppm)	0.011	0.038	0.024	0.007
O3 (ppm)	0.009	0.046	0.024	0.009
CO (ppm)	0.3	0.9	0.5	0.133

*Abbreviations: SD* standard deviation

The study examined whether the number of patients differed between the areas with high and low mean concentrations of PM_10_ for the entire study period. In other words, an independent sample t-test was conducted to compare the numbers of patients between the top 20% areas (top 29 administrative dongs) and the bottom 20% areas in terms of PM_10_ concentrations (bottom 29 administrative dongs) (Table 3). The results showed that the top 20% areas had significantly higher numbers of emergency patients for conjunctivitis (*p* < 0.01) and keratitis (*p* < 0.05) than the bottom 20% areas.

**Table 3:** Conjunctivitis or keratitis patients number differences between districts which stand for PM10 top 20% levels and districts which stand for PM10 bottom 20% levels from June, 2006 to December, 2014

		Visit Index^a^		*p* value
		Mean	SD	
Conjunctivitis	PM10 top 20% districts	0.8	0.5	< 0.01
	PM10 bottom 20% districts	0.3	0.7	
Keratitis	PM10 top 20% districts	2.4	0.9	< 0.05fIn multiple regression
	PM10 bottom 20% districts	1.4	1.7	

*Abbreviations: SD* standard deviation

^a^Visit Index – number of patients with conjunctivitis or keratitis / number of all patients × 1000

**Fig. 3:**
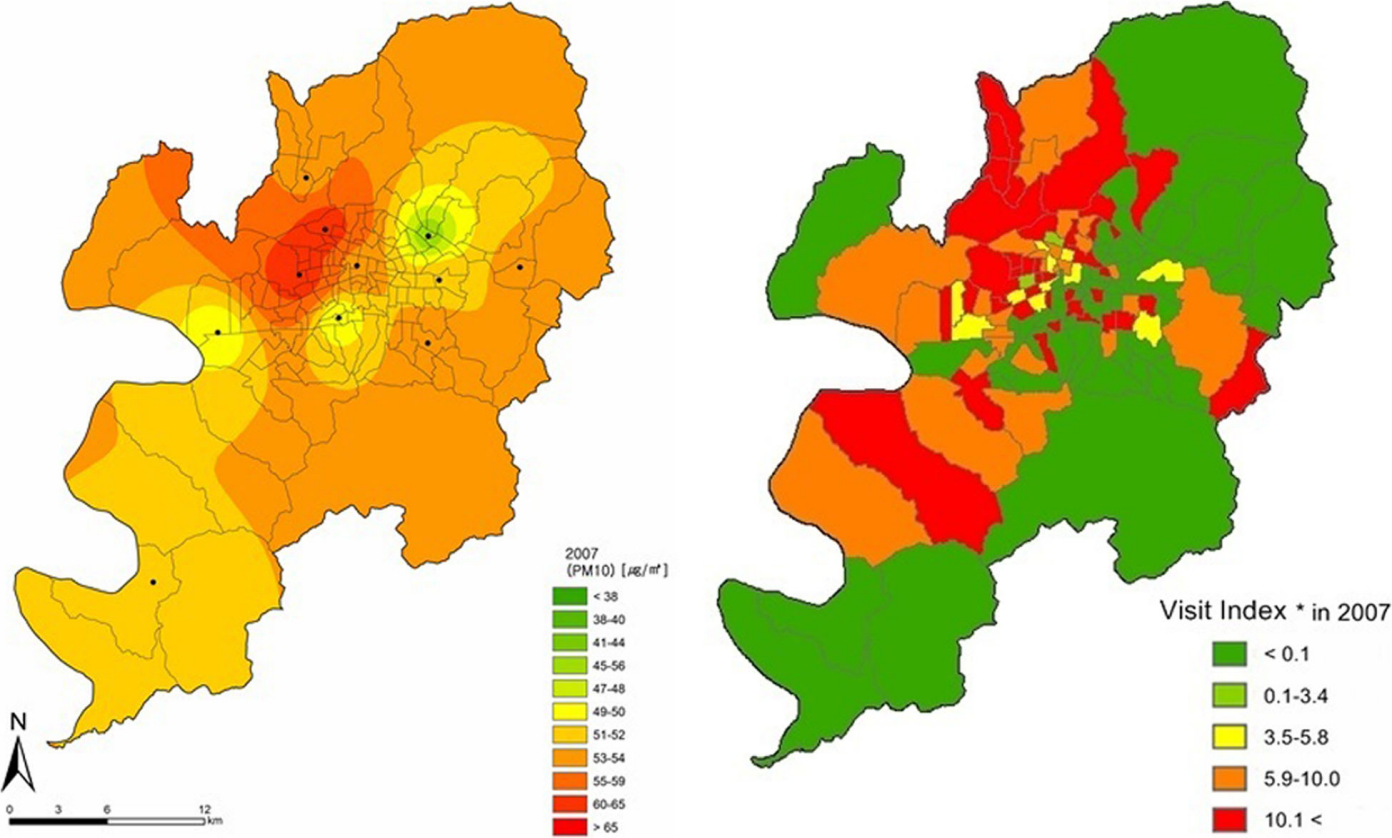
Spatial distribution of PM10 concentration and Visit Index of patients with conjunctivitis or keratitis in 2007. Visit Index – number of patients with conjunctivitis or keratitis / number of all patients × 1000

**Fig. 4:**
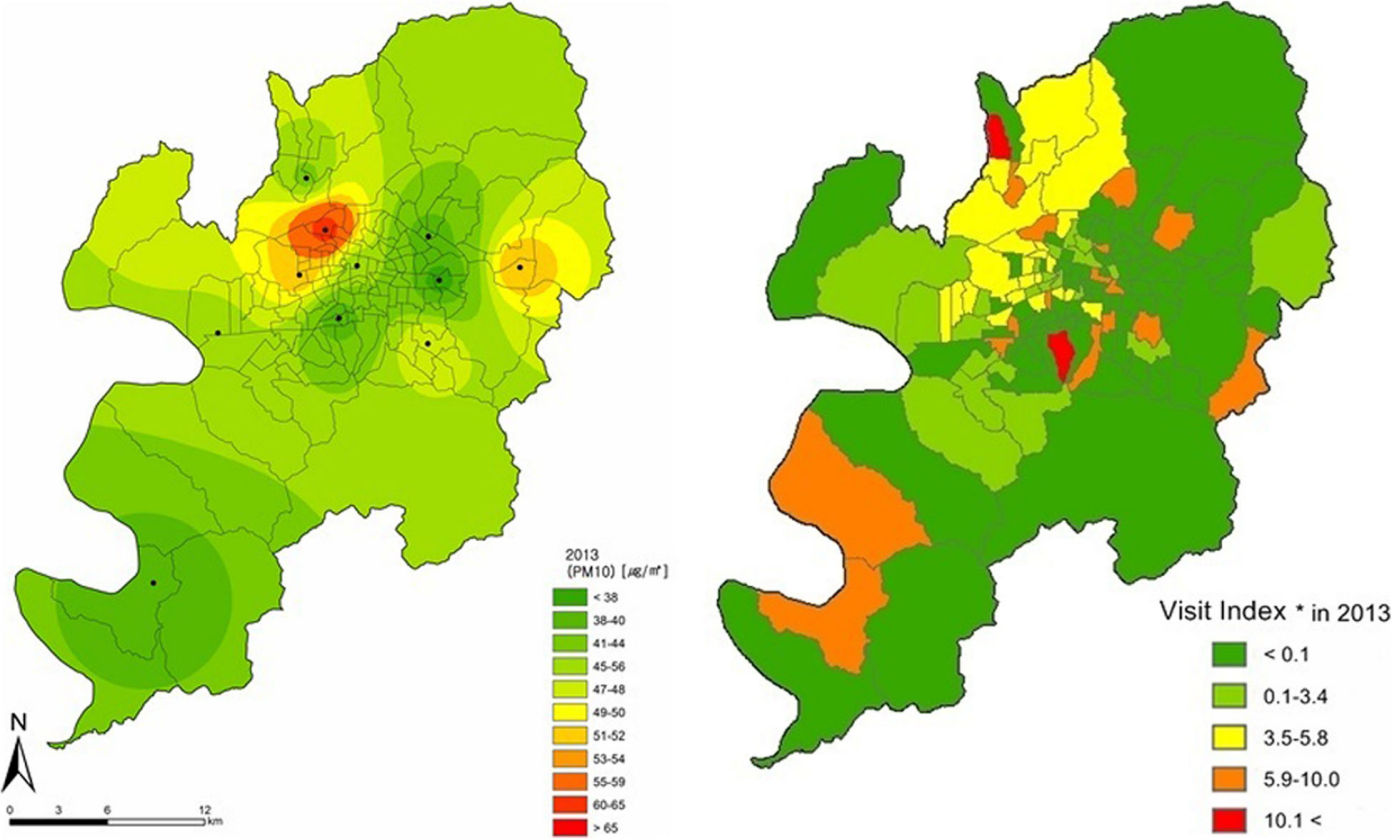
Spatial distribution of PM10 concentration and Visit Index of patients with conjunctivitis or keratitis in 2013. Visit Index – number of patients with conjunctivitis or keratitis / number of all patients × 1000

The distributions of PM_10_ and patients were mapped using ArcGIS, and compared by year (Figs. 3 and 4, Additional file 1: Figure S1, Additional file 2: Figure S2, Additional file 3: Figure S3, Additional file 4: Figure S4, Additional file 5: Figure S5, Additional file 6: Figure S6). Figs. 3 and 4 show the distributions of PM_10_ and patients in 2007 and 2013, respectively, which showed relatively clear regional differences during the study period. In 2007, PM_10_ concentrations were high in the northwestern region and the number of patients was also high in the northwest and some eastern parts, showing similar distributions between PM_10_ concentrations and patients. In 2013, PM_10_ concentrations were high in the northwest and some eastern parts, and the numbers of patients with conjunctivitis and keratitis were also high in the northwest, some eastern parts, and some southwestern parts, showing similar distributions between PM_10_ concentrations and patients.

**Fig. 5:**
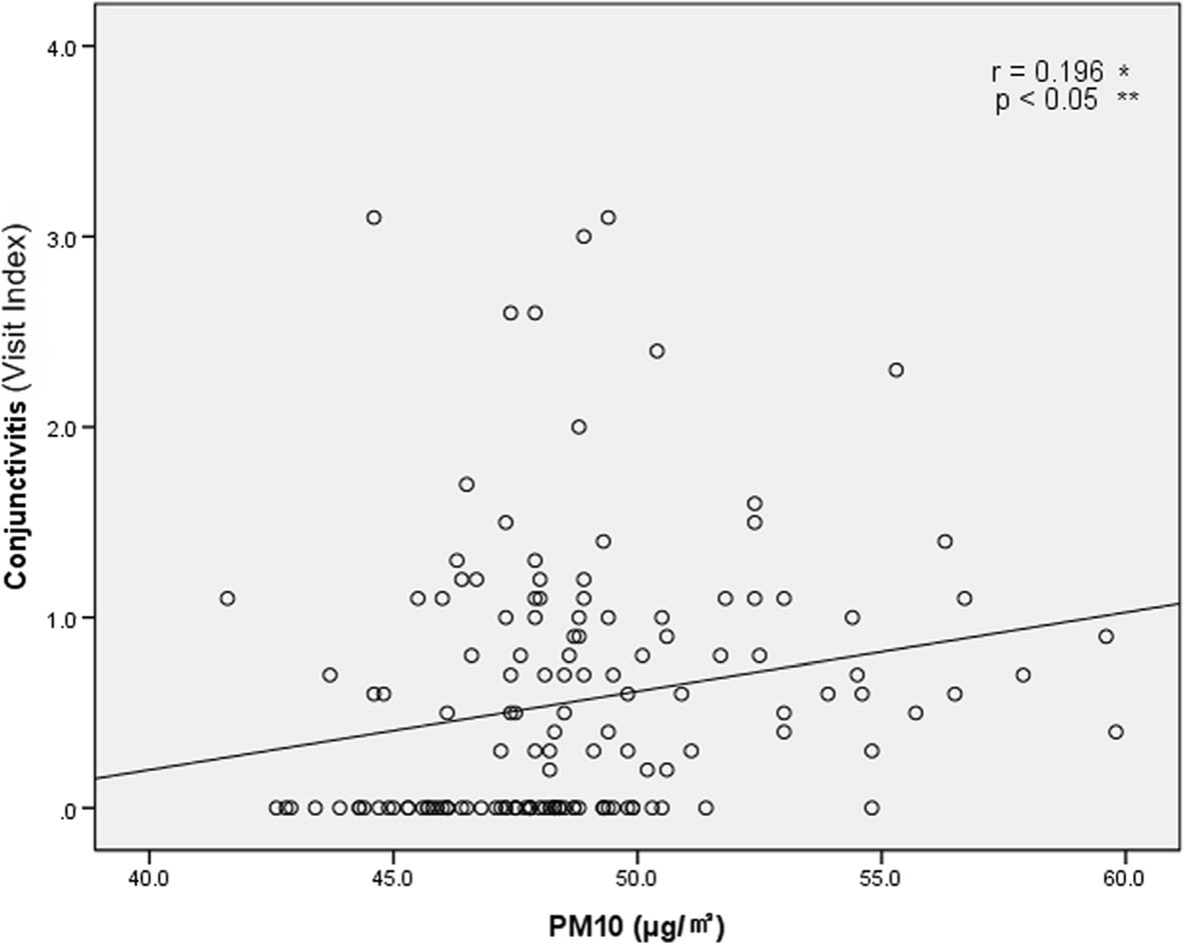
Dose-response relationship between PM10 concentration and Visit Index of patients with conjunctivitis from January, 2006 to December, 2014. Visit Index – number of patients with conjunctivitis or keratitis / number of all patients × 1000. *: Pearson’s correlation coefficient; **: statistical analysis by Pearson’s correlation analysis

**Fig. 6:**
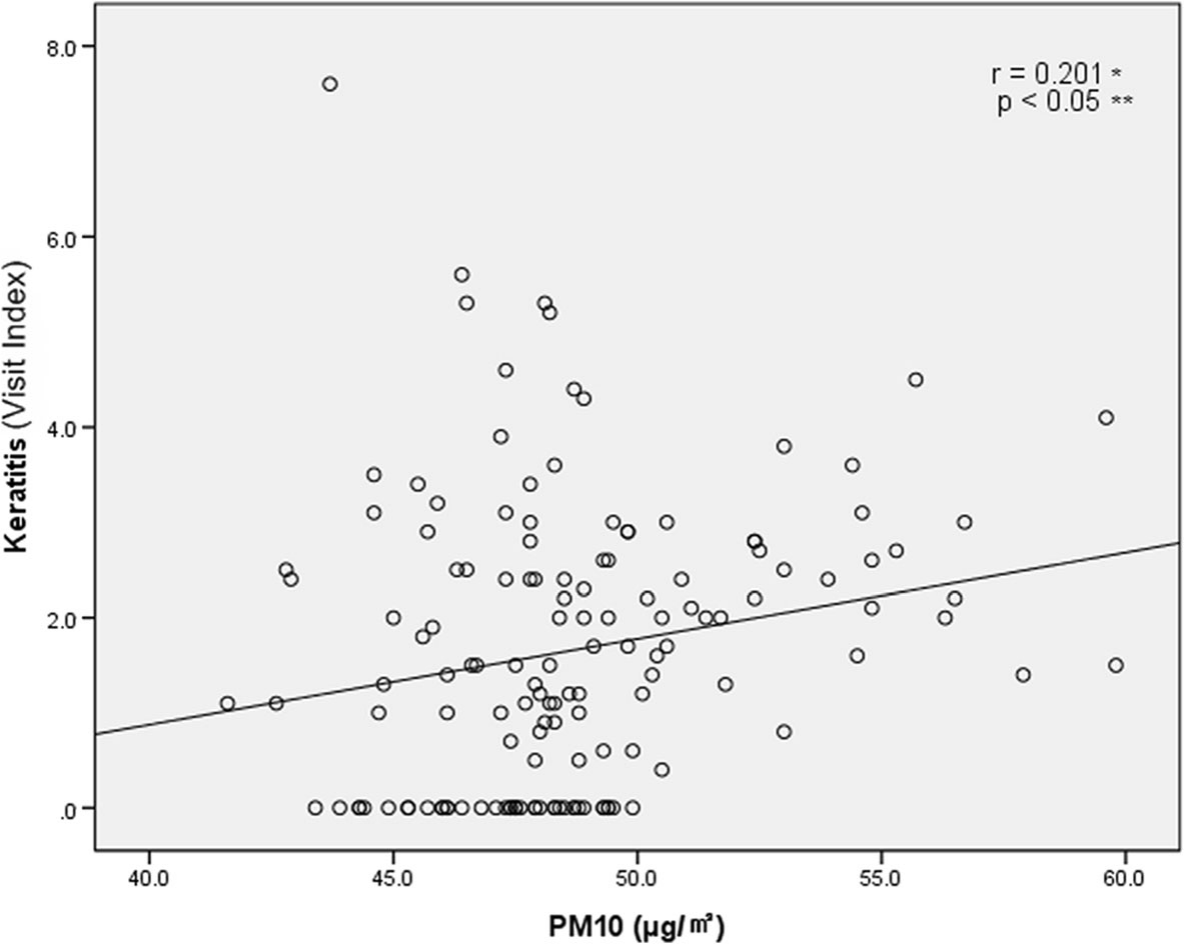
Dose-response relationship between PM10 concentration and Visit Index of patients with keratitis from January, 2006 to December, 2014. Visit Index – number of patients with conjunctivitis or keratitis / number of all patients × 1000. *: Pearson’s correlation coefficient; **: statistical analysis by Pearson’s correlation analysis

To determine the dose–response relationship between the PM_10_ concentration and the number of patients, scatter plots were plotted for each administrative dong. Figure 5 is a scatter plot showing the relationship between PM_10_ concentration and conjunctivitis, with a trend line with a positive slope. Figure 6 is a scatter plot showing the relationship between PM_10_ concentration and keratitis, also with a trend line with a positive slope.

A linear regression analysis was performed to determine the association between the concentration of PM_10_ and the numbers of conjunctivitis and keratitis patients (Table 4). In a simple linear regression analysis with PM_10_ only, the number of patients with conjunctivitis increased by 0.04 per 1000 patients in the emergency room and the number of patients with keratitis increased by 0.09 per 1000 patients in the emergency room when PM_10_ increased by 1 μg/m^3^, and the differences were statistically significant (*p* < 0.05). In multiple regression analysis with SO2, NO2, O3, and CO in addition to PM_10_, the number of patients with conjunctivitis increased by 0.04 per 1000 patients in the emergency room and the number of patients with keratitis increased by 0.10 per 1000 patients in the emergency room when PM_10_ increased by 1 μg/m^3^, and the differences were also statistically significant (*p* < 0.05).

**Table 4:** Linear regression analysis of the relationship between PM10 concentration and Visit Index^b^

	Unadjusted		Adjusted^c^	
	Coefficient	95% CI	Coefficient	95% CI
Conjunctivitis	0.040^a^	0.005–0.074	0.044^a^	0.001–0.088
Keratitis	0.089^a^	0.016–0.163	0.104^a^	0.010–0.198

*Abbreviations: CI* confidence interval

^a^*p*-value < 0.05

^b^Visit Index – number of patients with conjuctivitis or keratitis / number of all patients × 1000

^c^Adjusted for SO2, NO2, O3 and CO

## Discussion

This study investigated the relationship between the concentration of PM_10_ in Daegu and the numbers of conjunctivitis and keratitis patients who visited a university hospital emergency room, and found that the numbers of patients were significantly higher in the top 20% than in the bottom 20% areas in terms of PM_10_ concentration. The results of spatial analysis showed visual similarity between the distribution of PM_10_ concentration and the distribution of patients with conjunctivitis and keratitis for certain years during the study period. In addition, dose–response relationships were observed between PM_10_ concentration and the eye diseases, and the relationships were statistically significant even after controlling for leading gaseous air pollutants related to eye disease – sulfur dioxide (SO2), nitrogen dioxide (NO2), ozone (O3), and carbon monoxide (CO) [22].

Some cases of keratitis due to causes other than air pollutants such as PM_10_ (for example, photokeratitis from welding [23] and corneal ulcers from chemical burns [24]) may also be included in this study, and this could interfere with the reliability of the results. However, no patient was diagnosed with photokeratitis, so there was no need to consider it. In contrast, 53 patients (6.9%) were diagnosed with a corneal ulcer, so an analysis of keratitis excluding those cases was performed. The difference in the Visit Index between the top 20% districts for PM_10_ concentration and the bottom 20% districts was 0.9, which is statistically significant. In addition, linear regression showed that the unadjusted coefficient was 0.092 and the adjusted coefficient was 0.103; both are statistically significant (data are not shown). Therefore, the results before and after excluding the corneal ulcer patients were similar. In addition, there are various other causes of corneal ulcer besides chemical burn, which means that the influence of PM_10_ cannot be excluded in all cases. Therefore, the results of the analysis including the corneal ulcer cases are provided in this study.

Research on the health effects of particulate matter has often used the mean concentration of particulate matter for the entire area where the study participants resided as the particulate matter exposure level of the study participants [25]. However, air pollution assessments using only regional mean concentrations are likely to underestimate the localized increase in incidents of disease due to elevated concentrations near the sources of air pollutants [26, 27]. The health effects may be more pronounced around the source, but the effects may appear to be small if the mean concentration of the entire region is used [14]. Moreover, exposure to air pollution varies spatially within a city [28–31]. Thus, errors owing to classical exposure measurements can bias the results to null [32].

Regions selected for research on the health effects of particulate matter in the past often included metropolitan cities such as Beijing in China [3], Sao Paulo in Brazil [33], Seoul in Korea [34], and Texas in the U.S. [35] because the study findings had greater implications owing to the high population density of the cities, and these cities often show concentrations of particulate matter that are hazardous to health owing to a large concentration of industrial complexes and high traffic volume, thus generating a large amount of particulate matter. The city of Daegu, the study area in this work, also has general characteristics of metropolitan cities as it is one of the most populous cities in Korea, has a high concentration of industrial complexes, such as the Seongseo Industrial Park and the Third Industrial Park, and ranks fifth in terms of the length of road per area. However, Daegu is contrasted with other metropolitan cities in that it is a basin, which reduces air circulation.

Regarding the hospital selected for this study, Dongsan Medical Center is one of four tertiary hospitals in Daegu, and has high accessibility from various regions of the city owing to its location in the central district of Daegu. In addition, the distribution of emergency room visitors by medical specialty area for the hospital was similar to the national statistics based on the Yearbook of Emergency Medicine Statistics published by the National Emergency Medical Center, showing even distribution across specialties, which was another reason for selecting the hospital for this study.

Nucci et al. [4] reported that the incidence of conjunctivitis in pediatric populations was significantly higher in areas with high PM_10_ concentrations than in areas with low PM_10_ concentrations; Szyszkowicz et al. [22] reported a significant relationship between PM_2.5_ concentration and emergency room visits for conjunctivitis in both men (OR = 1.003, 95% CI: 1.000, 1.038) and women (OR = 1.017, 95% CI: 1.003, 1.031); Mimura et al. [36] showed a significant relationship (OR = 9.05) between PM_2.5_ concentration and allergic conjunctivitis during the period of May through July. In contrast, Gehring et al. [2] found no significant relationship between conjunctivitis and PM_10_ or PM_2.5_ concentrations in children and adolescents; Jiaxu et al. [37] found no significant relationship between the number of outpatient for allergic conjunctivitis and PM_10_ or PM_2.5_ concentrations.

Aerosol and soil bacteria are some of the major components of particulate matter. They may produce metabolites that affect the microbiome colonies of the ocular surface [38]. Changes in the microbiome of the eye can cause a secondary immunomodulating effect. Although the mechanism of this effect is not clearly known, it seems to involve oxidative stress, pro-inflammation, changes in intracellular proteins, stimulation of autonomic nervous system receptors, and inhibition of normal defense mechanisms [39]. This makes the eye vulnerable and may increase the likelihood of infectious eye diseases. In addition, particulate matter carries sources of infection from the natural environment to the eye. Therefore, particulate matter may directly cause infectious eye diseases. Moreover, these air pollutants accelerate the development of ocular inflammatory disease because they break the homeostasis of the tear film, ocular membrane, ocular surface, and eyelid margin of the eyeballs [22].

Previous studies have mostly shown that particulate matter can cause conjunctivitis. The present study showed that the prevalence of keratitis as well as conjunctivitis is associated with the concentration of PM_10_. In addition, the study found that the incidence of conjunctivitis and keratitis varies from region to region with the distribution of particulate matter, and is more frequent in areas with higher concentrations of particulate matter.

This study has several features that distinguish it from other studies.

The first distinctive feature is that the study area was the city of Daegu, which is situated in a basin. Daegu shares the characteristics of metropolitan cities, including large population, a large concentration of industrial complexes, and many roads, while having the distinctive feature of being in a basin, which reduces air circulation. In a basin, the surface temperature is often lower than the upper layer, which is called temperature inversion. In this case, the stability of the atmosphere weakens convection, and thus the radiation mist combines with the polluted air to create smog.

The second distinctive feature of this study is the systematic spatial analysis. When studying particles that vary in distribution across different spaces, such as particulate matter, there is a limitation in interpreting the study results accurately without understanding the spatial variation. In the present study, the study area of Daegu was divided into 143 administrative dongs and the concentration of particulate matter was obtained for each administrative dong. In particular, consideration of vertical variation is a large difference of this study from other studies that considered regional variation of particular matter, among which vertical variation was rarely considered [21]. The present study used contour lines considering the fact that the movement of particulate matter varies in actual travel time due to elevations in the terrain (e.g., hills and plane) as well as two-dimensional distances.

The present study also has several limitations.

First, most patients with eye disease visit the hospital through outpatient clinics rather than large hospital emergency rooms. The subjects of this study are expected to have had severe enough symptoms to visit a large hospital emergency room. Therefore, the results of this study can be only applicable to severe cases. Second, individual PM_10_ exposure concentrations were not measured. Most people spend the day sitting in transportation, working in the workplace, or remaining at home. The characteristics of indoor PM_10_ sources may be different from those of outdoor PM_10_ sources. However, one study has shown that indoor and outdoor concentrations of PM_10_ are highly correlated within individuals [40]. Therefore, ambient PM_10_ concentrations can be used as an indicator of exposure in studies involving health endpoints such as hospital visits. Third, because the study included only the patients who visited the emergency room of a single hospital as study participants, it was not possible to obtain information on the patients in the entire area of Daegu, and patients may have varied in terms of accessibility and preference regarding hospital choice. Therefore, the study tried to increase the generalizability by selecting the hospital located in the central district of Daegu. In addition, to control for the variation in the populations and hospital accessibility of individual administrative dongs, the Visit Index, which is the proportion of patients with conjunctivitis and keratitis in the total number of patients, instead of the number of the diseases, was used as the outcome variable. Fourth, subgroup analyses according to demographic characteristics, disease subtypes and other underlying diseases were not performed. Such analyses may offer more specific information on the effects of particulate matter because the susceptibility to particulate matter may vary according to gender [22] or age [31], and conjunctivitis and keratitis are classified into various subtypes, such as bacterial, fungal, and allergic types [41]. However, the number of study participants in this study was not sufficient to perform a subgroup analysis. In fact, most patients with conjunctivitis or keratitis rarely visit a hospital because they often monitor the progress at home while keeping their eyes clean or trying over-the-counter drugs [42]. Performing a special analysis on over a hundred administrative dongs require a large number of patients. In this study, the number of eligible study participants was smaller than expected and the spatial analysis was conducted on ocular surface disorders, including both conjunctivitis and keratitis. However, since the study results showed that both conjunctivitis and keratitis were significantly higher in the high PM_10_ concentration areas than in the low PM_10_ concentration areas, it is important to examine the pattern of the two diseases together. In addition, considering that both the conjunctiva and the cornea are the main constituents of the ocular surface and that particulate matter influences eye health primarily by breaking the homeostasis of the ocular surface and making it susceptible to infection [43], conjunctivitis and keratitis can be considered as a group of ocular surface disorders, within the context of the effects of particulate matter on health. Moreover, the fact that the study included only patients with conjunctivitis and keratitis who complained of symptoms severe enough to visit the emergency room suggests the possibility that the relationship between particulate matter concentration and eye diseases may be underestimated in this study. Conjunctivitis is more common in patients with allergic diseases such as asthma, allergic rhinitis, and atopic dermatitis [44], and keratitis is more common in diabetic patients [45]. However, there were not enough data on the history of underlying disease to be included in the analysis. Fifth, in the analysis on the spatial distribution of patients with keratitis and conjunctivitis, the spread of infectious eye disease among people was not considered. Some ocular diseases, such as epidemic keratoconjunctivitis and acute hemorrhagic conjunctivitis, are known to be caused by spread among people [46, 47]. However, it is a time-consuming and costly process to collect information on all the contacts between people. Furthermore, since particulate matter causes inflammation of the eyeballs primarily through the eye’s antioxidant defense mechanism, and carries a source of infection itself, the infection with the diseases can be related to exposure to particulate matter. Sixth, the registered addresses of study participants used in the study may be different from where they actually spend most of their time. The data of participants’ administrative dongs used in spatial analysis were obtained from the addresses provided at the time of their emergency room visit, but those in the same administrative dong may have individual differences in terms of the administrative dongs where they are exposed to particulate matter. For example, for a participant who spends most of the day at work located far from his or her residence, it would be more reasonable to use the concentration of particulate matter for the administrative dong for the workplace, rather than the administrative dong of the residence, as the amount of exposure. Even for those who spend most of their time in the administrative dong of their residences, the amount of exposure to particulate matter inevitably varies greatly between those who spend most of their time outdoors, such as children and adolescents, and those who stay mostly indoors, such as the elderly with illnesses. Although this study did not take into account all of these individual characteristics, the study has significance as an ecological study on the population as a whole. Seventh, there may be some errors in the classification of administrative dongs owing to the change of administrative districts during the study period. The study used the 2005 administrative district system with 143 administrative dongs in Daegu to calculate the PM_10_ concentrations for spatial analysis. However, some of the emergency room patients’ addresses included legal dongs instead of administrative dongs, and some administrative dongs were combined, split, or renamed during the study period. In addition, because a new address system called the street name address was designated as the legal address in July 2011, old addresses and new addresses have been used together. Since 2014, only the street name address has been used, which inevitably created errors. However, the study minimized the errors in the address conversion using Geocoding, which utilizes the coordinate system. Eighth, although we defined patient disease using the specific diagnostic code, the classification may have errors because the exact diagnosis of each case cannot be confirmed without the individual’s medical records [37].

Despite these limitations, this study has significant implications in that it investigated the relationship between PM_10_ concentration and eye disease using three-dimensional spatial analysis incorporating both distance and elevation, and showed the dose–response relationship as well as the specific role of PM_10_ concentration with other air pollutants controlled for.

## Conclusion

This study showed a spatial relationship between the concentration of PM_10_ and the emergency room visits of patients with conjunctivitis and keratitis in a region that has both the general characteristics of a metropolitan city and the specific feature of a basin. The findings of this study can be used as basic data for research on the effects of particulate matter on eye disease.

## Additional files

Additional file 1: **Figure S1.** Spatial distribution of PM10 concentration and Visit Index of patients with conjunctivitis or keratitis in 2008. Visit Index – number of patients with conjunctivitis or keratitis / number of all patients × 1000 (JPG 317 kb).

Additional file 2: **Figure S2.** Spatial distribution of PM10 concentration and Visit Index of patients with conjunctivitis or keratitis in 2009. Visit Index – number of patients with conjunctivitis or keratitis / number of all patients × 1000 (JPG 304 kb).

Additional file 3: **Figure S3.** Spatial distribution of PM10 concentration and Visit Index of patients with conjunctivitis or keratitis in 2010. Visit Index – number of patients with conjunctivitis or keratitis / number of all patients × 1000 (JPG 305 kb).

Additional file 4: **Figure S4.** Spatial distribution of PM10 concentration and Visit Index of patients with conjunctivitis or keratitis in 2011. Visit Index – number of patients with conjunctivitis or keratitis / number of all patients × 1000 (JPG 295 kb).

Additional file 5: **Figure S5.** Spatial distribution of PM10 concentration and Visit Index of patients with conjunctivitis or keratitis in 2012. Visit Index – number of patients with conjunctivitis or keratitis / number of all patients × 1000 (JPG 304 kb).

Additional file 6: **Figure S6.** Spatial distribution of PM10 concentration and Visit Index of patients with conjunctivitis or keratitis in 2014. Visit Index – number of patients with conjunctivitis or keratitis / number of all patients × 1000 (JPG 298 kb).

## Abbreviations

CO: Carbon monoxide
IDW: Inverse Distance Weighted
NO2: Nitrogen dioxide
O3: Ozone
PM: Particulate matter
SO2: Sulfur dioxide
