# Supplementary figures and images for: Spatial analysis between particulate matter and emergency room visits for conjunctivitis and keratitis
Source: Ann Occup Environ Med. 2018 Jun 11;30:41. doi: 10.1186/s40557-018-0252-x (PMC5996503; doi:10.1186/s40557-018-0252-x)

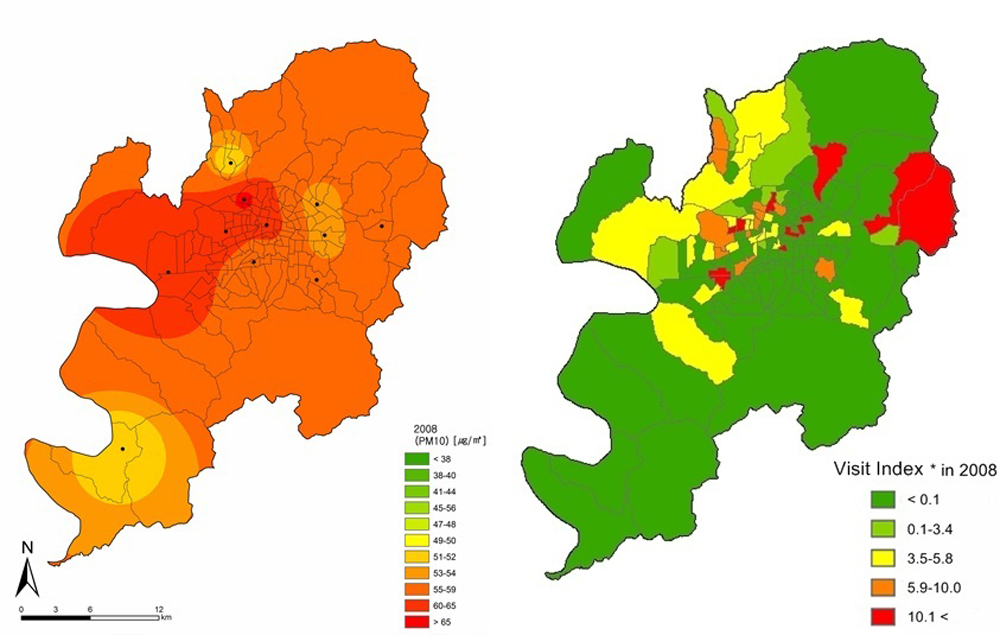

Supplement: Supplementary file 1 — Figure S1. Spatial distribution of PM10 concentration and Visit Index of patients with conjunctivitis or keratitis in 2008. Visit Index – number of patients with conjunctivitis or keratitis / number of all patients × 1000 (JPG 317 kb). [file 40557_2018_252_MOESM1_ESM.jpg]

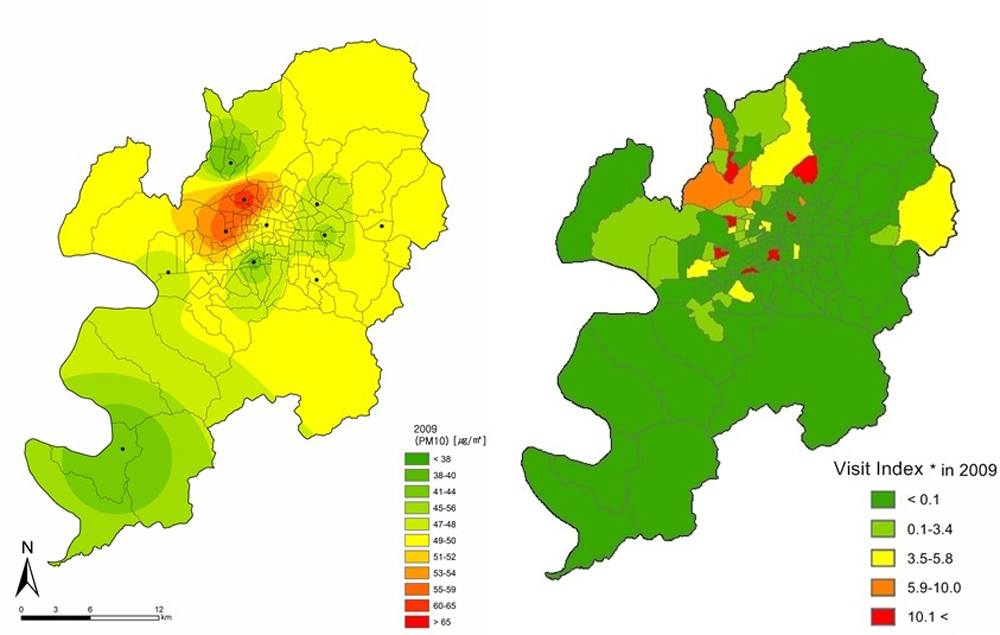

Supplement: Supplementary file 2 — Figure S2. Spatial distribution of PM10 concentration and Visit Index of patients with conjunctivitis or keratitis in 2009. Visit Index – number of patients with conjunctivitis or keratitis / number of all patients × 1000 (JPG 304 kb). [file 40557_2018_252_MOESM2_ESM.jpg]

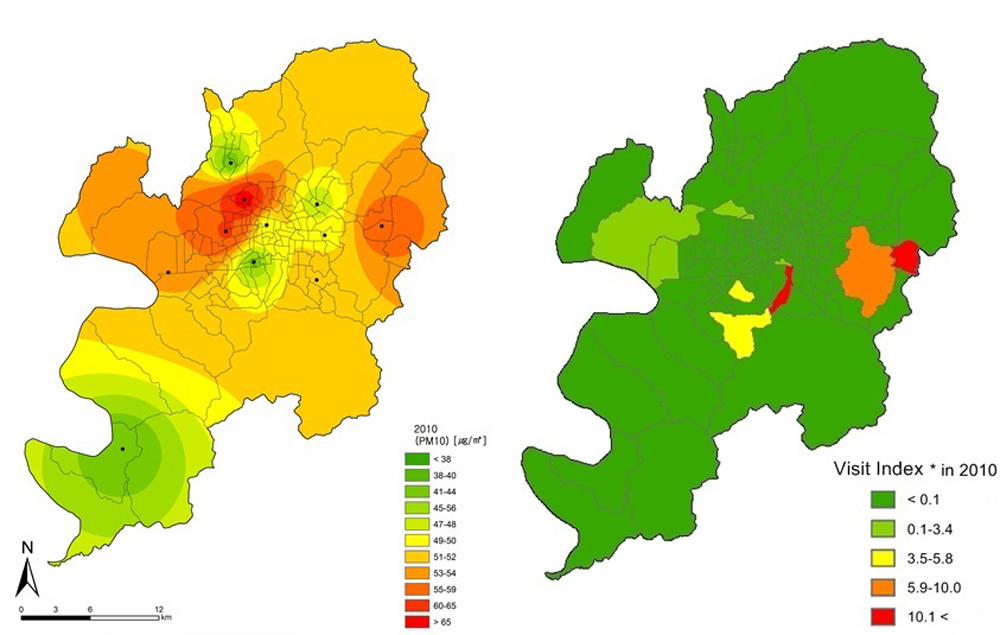

Supplement: Supplementary file 3 — Figure S3. Spatial distribution of PM10 concentration and Visit Index of patients with conjunctivitis or keratitis in 2010. Visit Index – number of patients with conjunctivitis or keratitis / number of all patients × 1000 (JPG 305 kb). [file 40557_2018_252_MOESM3_ESM.jpg]

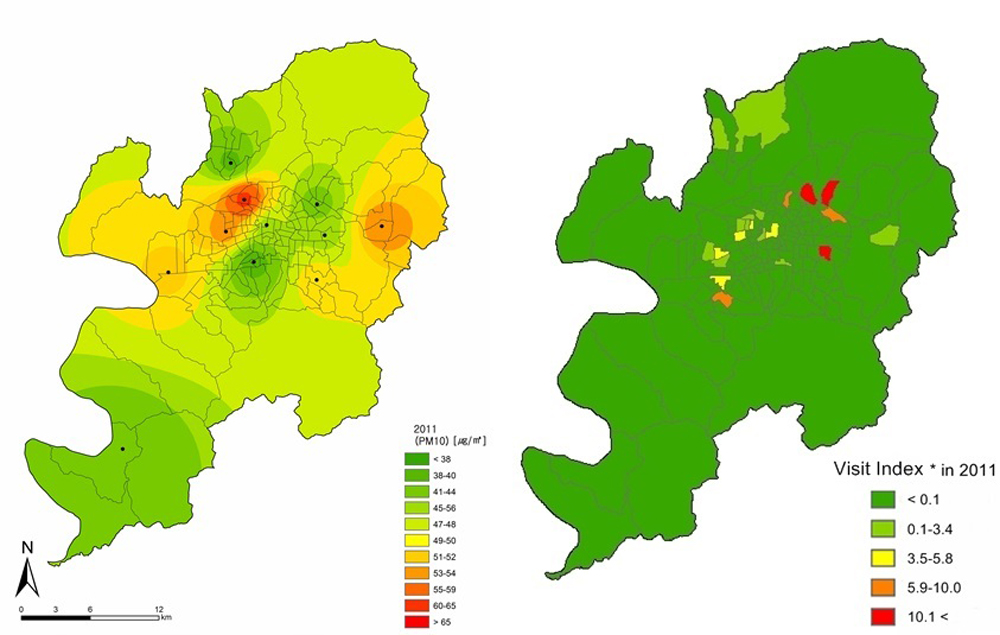

Supplement: Supplementary file 4 — Figure S4. Spatial distribution of PM10 concentration and Visit Index of patients with conjunctivitis or keratitis in 2011. Visit Index – number of patients with conjunctivitis or keratitis / number of all patients × 1000 (JPG 295 kb). [file 40557_2018_252_MOESM4_ESM.jpg]

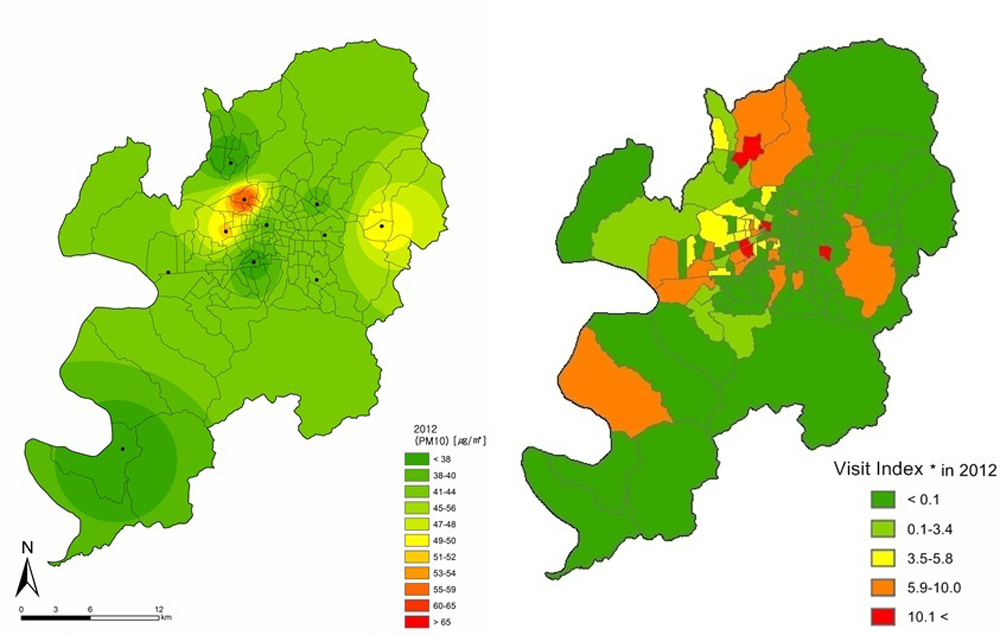

Supplement: Supplementary file 5 — Figure S5. Spatial distribution of PM10 concentration and Visit Index of patients with conjunctivitis or keratitis in 2012. Visit Index – number of patients with conjunctivitis or keratitis / number of all patients × 1000 (JPG 304 kb). [file 40557_2018_252_MOESM5_ESM.jpg]

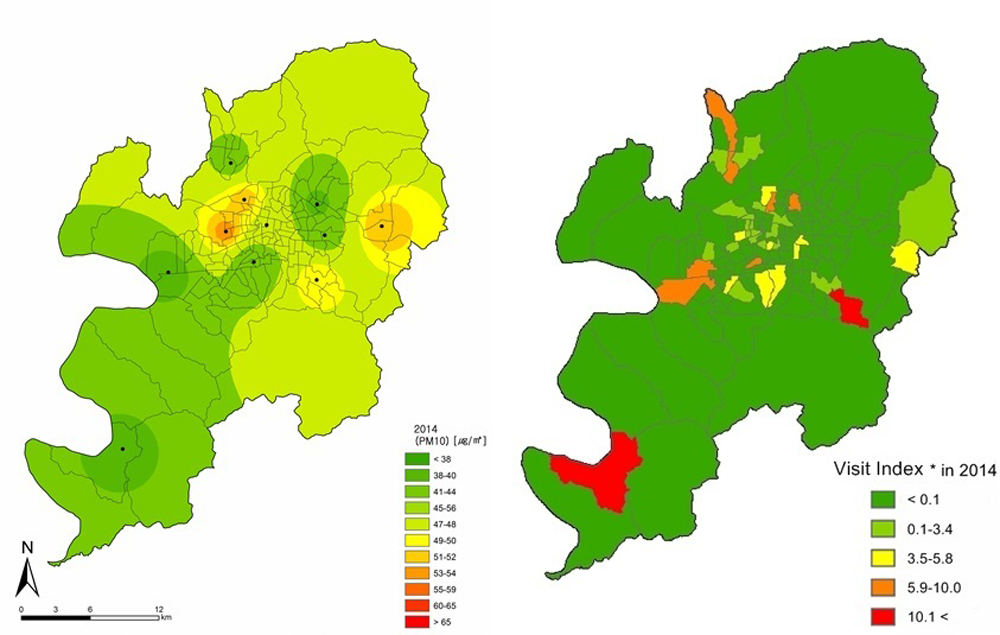

Supplement: Supplementary file 6 — Figure S6. Spatial distribution of PM10 concentration and Visit Index of patients with conjunctivitis or keratitis in 2014. Visit Index – number of patients with conjunctivitis or keratitis / number of all patients × 1000 (JPG 298 kb). [file 40557_2018_252_MOESM6_ESM.jpg]
